# A Novel Control Strategy for Soft Open Point to Address Terminal Voltage Violations and Load Rate Imbalance in Low-Voltage Power Distribution Station Areas

**DOI:** 10.3390/s24061976

**Published:** 2024-03-20

**Authors:** Zhichun Yang, Huaidong Min, Fan Yang, Yang Lei, Xiangling He, Haichang Sun, Xu Tang

**Affiliations:** 1 Electric Power Research Institute, State Grid Hubei Electric Power Co., Ltd., Wuhan 430077, China; yangzc8@hb.sgcc.com.cn (Z.Y.); minhd@hb.sgcc.com.cn (H.M.); yangf81@hb.sgcc.com.cn (F.Y.); leiyang@hb.sgcc.com.cn (Y.L.); 2 Hubei Key Laboratory of Power Equipment & System Security for Integrated Energy, Wuhan 430072, China; 2019302070297@whu.edu.cn (X.H.); 2019302070148@whu.edu.cn (H.S.); 3 School of Electrical Engineering and Automation, Wuhan University, Wuhan 430072, China

**Keywords:** low-voltage power distribution station area (DSA), load rate imbalance, renewable energy consumption, soft open point (SOP), sensor networks, voltage regulation

## Abstract

In low-voltage power distribution station areas (DSAs), sensor devices and communication networks are often inadequate. Therefore, the control strategies mainly used for soft open points (SOPs) based on global information in medium-voltage distribution networks are difficult to be directly applied to low-voltage DSAs. This paper proposes a novel control strategy for SOP that only requires collecting the local information of SOP and the load rate of transformers. It aims to address the issues faced of voltage violations at the end of feeders and the load rate imbalance among adjacent DSAs under the current high penetration of renewable energy sources. In this paper, first, a sensor network consisting of sensor devices located at the transformers and each port of the SOP is introduced for information collection. Then, based on the sensitivity relationship between the node voltage and the injected power, considering capacity and voltage safety constraints, the adjustable range of the active power output for each port of the SOP is derived. According to this range, the operating states of the DSAs are categorized into four scenarios. For each scenario, the adjustment amount of SOP output power is determined to achieve comprehensive regulation of terminal voltage and load rate of all DSAs interconnected by SOP. Finally, the effectiveness of the proposed strategy is verified based on a simulation model of three flexible interconnected DSAs established in MATLAB/Simulink.

## 1. Introduction

A low-voltage power distribution station area (DSA) is the power supply area of a distribution transformer (usually 35 kV/380 V or 10 kV/380 V). The low-voltage DSA is a power supply unit directly serving users, and its operational status directly affects the economic efficiency of the power grid and the quality of the user power supply [1]. Currently, to reduce the level of carbon emissions, the installed proportion of renewable energy sources (RESs) has gradually been increased, and various distributed generators (DGs) represented by photovoltaic (PV) are being connected to low-voltage power DSAs in large quantities. Due to the fluctuating output of DGs and the incomplete match between output and the local load demand, DSAs with high PV penetration face challenges such as reverse power flow and voltage violations [2]. Literature [3,4,5] indicates that the voltage violation caused by mismatched source–load power is one of the main factors limiting the consumption capacity of RESs in distribution networks. This is because in the absence of energy transfer pathways, the output power of DG units that cannot be locally consumed in DSAs can only be fed back to the higher-level grid. When this reverse power flow causes the voltage to exceed the upper limit, measures like curtailing wind or solar power are necessary to ensure that the voltage remains within the permissible range, thereby limiting the increase in DG penetration rate. Also, the voltage at the feeder end is the most sensitive to fluctuations in DG output power, making voltage violations more likely and affecting normal power consumption by users [2,6]. Therefore, studying the regulation of voltage violations at the end of feeders in DSAs is of significant importance for enhancing the distribution network’s capacity to accommodate DGs.

Additionally, the problem of load rate imbalance among adjacent DSAs is becoming more prominent, affecting the economic efficiency of its operations. On the one hand, due to the increasing penetration rate of DG and its uneven distribution across different DSAs, certain DSAs with high DG penetration rates often operate with underutilized capacity, resulting in low-efficiency operation of their distribution transformers and economic inefficiencies [7]. On the other hand, some DSAs experience seasonal load fluctuations due to electrical projects and tourism development. During peak demand seasons, these DSA transformers may experience short-term heavy loads or even overloads, causing high stress to transformers and requiring the deployment of mobile energy storage devices or emergency diesel generators to cope with short-term peak electricity demand [1]. If the surplus capacity of transformers among adjacent DSAs can be shared, transferring part of the load from heavy-loaded DSAs to low-loaded DSAs, the utilization rate of transformer capacity in low-loaded DSAs can be improved, and the supply pressure on heavy-loaded DSAs can be alleviated, effectively enhancing the economic efficiency of DSAs [8].

The soft open point (SOP) is a new type of multi-port power electronic equipment used to achieve flexible interconnection among feeders. It can facilitate fast and precise active power transfer among feeders and provide reactive power compensation [9]. As the issues of voltage violation and unbalanced load rate faced by the DSAs are fundamentally caused by the uneven spatial distribution of source–load, using SOP for flexible interconnection among DSAs can achieve power transfer in space, thus simultaneously addressing the issues. This effectively improves the consumption capacity of DGs, enables dynamic expansion of DSA supply capacity, improves operational economic efficiency, and possesses advantages over traditional solutions that focus on a single issue. Traditional solutions for voltage violation mainly include on-load voltage regulation strategies [10], reactive power regulation strategies [11], and active power regulation strategies [12]. Although the on-load voltage regulation strategy based on adjusting the transformer tap gear is low in cost, the regulation quantity is discontinuous and cannot be adjusted frequently, which makes it difficult to deal with the problem of voltage violation at the end of feeders in DSAs caused by frequent fluctuation of DG output. The reactive power regulation strategy based on reactive power compensation can achieve continuous and rapid voltage regulation using devices like static var generators (SVG). However, due to the higher resistance-to-reactance ratio (r/x) of low-voltage distribution feeders, utilizing SOP based on the active–reactive coordinated voltage regulation has higher efficiency than an SVG with equivalent capacity. What is more, it can further achieve the goal of load rate balancing among DSAs in addition to voltage regulation. As for the active power regulation strategies, there are two methods available. One is to reduce the output of DGs, which will directly reduce the economic benefits of DGs. The other is to configure an energy storage system (ESS) to prevent voltage violation by absorbing DG output that cannot be consumed locally. Unfortunately, the investment in ESS is high, and it is not economical to configure it only for a single station area. However, the use of SOP for flexible interconnection among DSAs allows the transfer of DG output that cannot be locally consumed to adjacent DSAs for joint consumption, which achieves voltage regulation while also achieving load rate balancing.

Currently, there are many research studies on the application of SOP in medium-voltage distribution networks. Literature [13] shows studies conducted on the working principles of SOP in distribution networks. Ref. [14] establishes an optimized scheduling model including SOP for active distribution networks, to coordinate and control controllable resources within the system to achieve optimal power flow and suppress voltage violations. Ref. [15], based on [14], further considers real-time voltage control and establishes an optimization model considering the Q-V droop curve of SOP, but does not consider the goal of load rate balancing. Ref. [16] suggests that adopting a centralized control approach will impose significant communication pressure. It considers using a distributed approach to address the optimization scheduling problem and establishes a distributed optimization scheduling model. Ref. [17], based on [16], considers local voltage control issues and establishes an optimization scheduling model that combines distributed and local control with SOP. Ref. [18] proposes combining ESS with SOP, enabling SOP to have flexible control capabilities in both time and space dimensions. Ref. [19], aiming to increase the penetration rate of renewable energy while ensuring voltage within limits, integrates SOP, PV, and thermal ESS into the distribution network. It proposes a double-layer optimization model to coordinate the operation of various controllable devices. Ref. [20], with the goal of increasing the penetration rate of renewable energy, comprehensively considers various flexible and controllable resources in a mixed AC/DC distribution network. It establishes a two-stage optimization model for the day-ahead and intra-day periods, maintaining the optimal operation of the distribution network under high renewable energy penetration. Ref. [21] comprehensively considers multiple controllable devices, including SOP, PV inverters, and reactive power devices, to coordinate and cooperate in solving the voltage violation issue in medium-voltage distribution networks.

The above studies on the application of SOP in the distribution network are mostly aimed at the medium-voltage distribution network. Global information is needed to control SOP to achieve voltage regulation and improve DG penetration by establishing an optimal scheduling model. These studies provide valuable insights for the application of SOP in low-voltage DSAs. However, there are certain limitations. On the one hand, the sensor devices and communication networks in low-voltage DSAs are not as complete and robust as those in medium-voltage distribution networks [22]. Applying the optimal scheduling models developed for medium-voltage distribution networks may face challenges related to information collection and transmission in low-voltage DSAs. On the other hand, even in some demonstration projects with interconnected multiple DSAs having a globally reliable communication network for optimal scheduling, the widely proposed framework for multi-timescale optimization scheduling models typically follows a day-ahead scheduling and intraday rolling optimization. Constrained by the collection, transmission, and processing of network-wide information, the time interval between two optimization scheduling instances is generally around 15 min. The voltage violations caused by fluctuating source–load power within this time interval need to wait for the next scheduling to issue corrective instructions, resulting in a less responsive solution.

Considering these limitations, this paper proposes a novel control strategy for SOP that relies only on SOP local information and transformer load rate to address voltage violations at the end of feeders and load rate imbalance among adjacent DSAs. On the one hand, in DSAs where global reliable communication conditions are not available, the proposed strategy does not require the collection of information from all nodes in the network and can focus on addressing the problem of terminal voltage violations at the end of feeders among adjacent DSAs, achieving the goal of substantially load rate balancing of DSAs. On the other hand, in DSAs with globally reliable communication, this strategy can serve as a complementary measure to global optimal scheduling. It addresses issues related to voltage violations at the end of DSAs or unbalanced load rates at a sub-second timescale, responding rapidly to fluctuations in source–load power. Based on the proposed strategy, the capacity of low-voltage DSAs to accommodate DGs can be effectively enhanced, and the operational economy of distribution transformers can be improved.

The main contributions of this research work are as follows:

1. A novel control strategy for SOP is proposed which relies solely on SOP local information and the load rate of transformers. This strategy can effectively address issues of voltage violations at the end of feeders and load rate imbalance among adjacent DSAs. It does not require reliance on a global sensor network and communication network, making it highly applicable in low-voltage distribution networks.
2. Due to its independence from gathering global information for centralized decision-making, this strategy can respond on shorter time scales. As a result, it can serve as a complementary approach to hourly optimization scheduling strategies.

The remainder of this paper is organized as follows. Section 2 introduces the topology, sensor network, and voltage regulation principles of flexible interconnected low-voltage DSAs; Section 3 gives the proposed control strategy; Section 4 presents a case study to demonstrate the effectiveness of the proposed strategy; Section 5 summarizes the whole paper.

## 2. Topology, Sensor Network, and Voltage Regulation Principles of Flexible Interconnected Low-Voltage DSAs

First, this section introduces the basic topology of the flexible interconnection DSAs, the sensor network, and the communication architecture required for the proposed strategy. Then, the principle of voltage regulation is analyzed as the basis for the strategy proposed in the following text.

### 2.1. Topology and Sensor Network of Flexible Interconnected Low-Voltage DSAs

**Figure 1:**
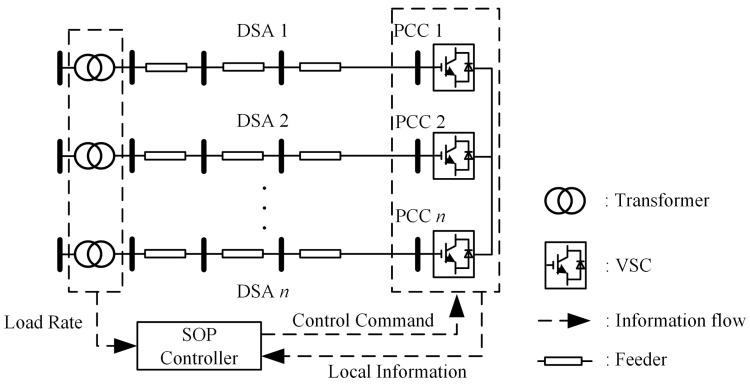
Flexible interconnected low-voltage DSAs based on SOP and its sensor network.

The topology and sensor network of flexible interconnected low-voltage DSAs are shown in Figure 1. The SOP consists of multiple back-to-back voltage source converters (VSCs), with each VSC AC side connected to the feeder end of a low-voltage DSA. The DC sides of all VSCs are interconnected. Due to the fact that low-voltage distribution networks often do not have sensor devices installed at all nodes, it is not possible to collect information from all nodes. However, in order to ensure the normal operation of the equipment, sensors are usually installed at the transformer and each port of the SOP. Therefore, we propose to construct a simple sensor network based on the existing sensors at the transformer and SOP ports to obtain the load rate information of the transformer and local information of SOP, namely, the voltage value at the point of common connection (PCC) between SOP and the feeder, and the initial power output of SOP. The proposed strategy relies solely on this information to adjust the output power of each VSC, addressing the issues of terminal voltage violations and load rate imbalance. When all voltages at PCCs are within limits, and the load rate among DSAs is balanced, the strategy does not change the current output power of VSCs. When it is detected that voltages at PCCs exceed limits or the load rate among DSAs is highly unbalanced, the strategy responds immediately, adjusts the output power of VSCs to ensure voltages at the PCCs do not exceed the limits, and balances the load rate among DSAs.

### 2.2. Voltage Regulation Principles at the End of Feeders Based on SOP

**Figure 2:**
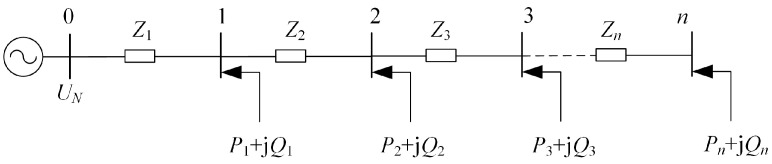
Schematic diagram of a distribution feeder.

Voltage regulation at the end of DSAs through SOP is achieved by changing the injected power to the connected node. When transferring active power between DSAs to balance the load rates, it is also necessary to prevent voltage violations caused by power transfer. So, it is necessary to clarify the sensitivity relationship between node injected power and node voltage. For a distribution feeder as illustrated in Figure 2, assuming its rated voltage is *U*_N_, the net injected power at node *i* is *P_i_* + j*Q_i_*, the voltage at node *i* is *U_i_*, and the impedance between node *i −* 1 and node *i* is *Z_i_* = *R_i_* + j*X_i_*.

For the distribution feeder shown in Figure 2, the deviation Δ*U_im_* between the voltage of node *i* and the rated voltage due to the injection power at node *m* can be represented by Equation (1) [23]:

(1) $$\Delta U_{im}=\left\{\begin{array}{l} \frac{1}{U_{N}}\sum_{k=1}^{m}Z_{k}\left(P_{m}-jQ_{m}\right)\;m<i\\ \frac{1}{U_{N}}\sum_{k=1}^{i}Z_{k}\left(P_{m}-jQ_{m}\right)\;m\geq i \end{array}\right.$$

*U*_N_ represents the rated voltage of DSAs, *P_m_*, and *Q_m_* are the active and reactive power injected by node *m*, and *Z_k_* represents the impedance between node *k* and node (*k* + 1), where *Z_k_ = R_k_* + *X_k_*.

When node *m* injects a disturbance power with a magnitude of Δ*P_m_* + jΔ*Q_m_*, the perturbation of Δ*U_im_* is given by Equation (2):

(2) $$\Delta {\tilde{U}}_{im}=\left\{\begin{array}{l} \frac{1}{U_{N}}\left(\Delta P_{m}\sum_{k=1}^{m}R_{k}+\Delta Q_{m}\sum_{k=1}^{m}X_{k}\right)+j\frac{1}{U_{N}}\left(\Delta P_{m}\sum_{k=1}^{m}X_{k}-\Delta Q_{m}\sum_{k=1}^{m}R_{k}\right),\;m<i\\ \frac{1}{U_{N}}\left(\Delta P_{m}\sum_{k=1}^{i}R_{k}+\Delta Q_{m}\sum_{k=1}^{i}X_{k}\right)+j\frac{1}{U_{N}}\left(\Delta P_{m}\sum_{k=1}^{i}X_{k}-\Delta Q_{m}\sum_{k=1}^{i}R_{k}\right),\;m\geq i \end{array}\right.$$

The voltage level is 380 V in the low-voltage DSAs, so the imaginary part of $\Delta {\tilde{U}}_{im}$ can be neglected [24]. Defining *S_Pim_* and *S_Qim_* as the sensitivity coefficients of the voltage fluctuation at node *i* to changes in active and reactive power injection at node *m*, the expressions for *S_Pim_* and *S_Qim_* can be obtained from Equation (3):

(3) $$\begin{array}{l} S_{Pim}=\left\{\begin{array}{l} \frac{1}{U_{N}}\sum_{k=1}^{m}R_{k},\;m<i\\ \frac{1}{U_{N}}\sum_{k=1}^{i}R_{k},\;m\geq i \end{array}\right.\\ S_{Qim}=\left\{\begin{array}{l} \frac{1}{U_{N}}\sum_{k=1}^{m}X_{k},\;m<i\\ \frac{1}{U_{N}}\sum_{k=1}^{i}X_{k},\;m\geq i \end{array}\right. \end{array}$$

The physical meaning of *S_Pim_* and *S_Qim_* is that, near the rated voltage value, a unit change in the injected active and reactive power at node *m* will, respectively, cause a change of *S_Pim_* and *S_Qim_* in the voltage at node *i*. Therefore, when voltage violations occur at the end of DSAs, it can adjust the injected power at the terminal node by transferring active power through SOP and providing reactive compensation, thereby achieving voltage regulation at the terminal node. For example, when the terminal node voltage *U* falls below the lower limit allowed $\underline{U}$, adjusting the injected power of SOP based on Equation (4) can eliminate the voltage violation:

(4) $$\underline{U}-U=S_{Pnn}\cdot \Delta P_{\mathrm{VSC}}+S_{Qnn}\cdot \Delta Q_{\mathrm{VSC}}$$

where *S_Pnn_* and *S_Qnn_* are the sensitivity coefficients of the injected active and reactive power at node *n* to the voltage value at node *n*. Δ*P*_VSC_ and Δ*Q*_VSC_ represent the adjustments in active and reactive power injected by the VSC connected to node *n*. The specific values of Δ*P*_VSC_ and Δ*Q*_VSC_ are provided by the proposed strategy.

## 3. Proposed Strategy for Addressing Terminal Voltage Violations and Load Rate Imbalance

As discussed earlier, when the sensitivity relationship between the adjustment of the voltage at the terminal node and the adjustment of the injected power of the VSC connected to the terminal node is known, regulating terminal voltage violations can be achieved by adjusting the injected power of the VSC. At the same time, balancing the load rates is also achieved by directly changing the injected active power of each VSC into each DSA. This chapter primarily discusses how to adjust the power injected by the VSC to address the voltage violations at the end of DSAs and the unbalanced load rates among DSAs.

### 3.1. The Range of Adjustable Active Power Output of Each VSC

Due to the energy conservation requirement of the SOP, adjusting the active power output of one VSC will affect the active power output of the other VSCs. Therefore, it is necessary to ensure that the adjustment of the active power output for each VSC remains within an acceptable range during the adjustment process. The range of adjustable active power output of the VSC $\Omega_{P}$ is defined as shown in Equation (5):

(5) $$\Omega_{P}=\left\{\Delta P_{\mathrm{VSC}}|\Delta P_{\mathrm{VSC},\min}\leq \Delta P_{\mathrm{VSC}}\leq \Delta P_{\mathrm{VSC},\max}\right\}$$

Δ*P*_VSC,min_ and Δ*P*_VSC,mx_ are the maximum and minimum values of the VSC acceptable output range.

**Figure 3:**
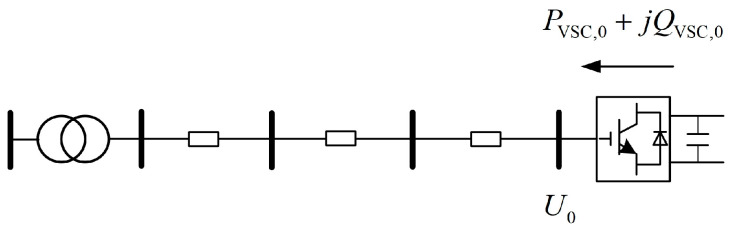
Schematic diagram of an interconnected DSA.

Consider a DSA as shown in Figure 3, where the injection of power by the VSC toward the feeder is defined as the positive direction. Suppose the voltage at the terminal node before adjusting the VSC injection power is denoted as *U*_0_, then the initial power output of the VSC is *P*_VSC,0_ + j*Q*_VSC,0_.

The $\Omega_{P}$ constrained by the upper and lower limits of the terminal node voltage can be described by Equation (6):

(6) $$\left\{\begin{array}{l} U_{0}+S_{P}^{\mathrm{VSC}}\cdot \Delta P_{\mathrm{VSC}}+S_{Q}^{\mathrm{VSC}}\cdot \Delta Q_{\mathrm{VSC}}\geq \underline{U}\\ U_{0}+S_{P}^{\mathrm{VSC}}\cdot \Delta P_{\mathrm{VSC}}+S_{Q}^{\mathrm{VSC}}\cdot \Delta Q_{\mathrm{VSC}}\leq \bar{U} \end{array}\right.$$

where $S_{P}^{\mathrm{VSC}}$ and $S_{Q}^{\mathrm{VSC}}$ are the sensitivity coefficients of the active and reactive power of the terminal node voltage, respectively; Δ*P*_VSC_ and Δ*Q*_VSC_ are the adjustments of the active and reactive power injected by VSC, respectively; $\bar{U}$ and $\underline{U}$ are the upper and lower limits of the voltage, respectively.

The $\Omega_{P}$ constrained by the capacity of VSC can be described by Equation (7) [25]:

(7) $${\left(P_{0}+\Delta P_{\mathrm{VSC}}\right)}^{2}+{\left(Q_{0}+\Delta Q_{\mathrm{VSC}}\right)}^{2}\leq {\left(S_{\mathrm{VSC}}\right)}^{2}$$

where $S_{\mathrm{VSC}}$ is the capacity of VSC.

**Figure 4:**
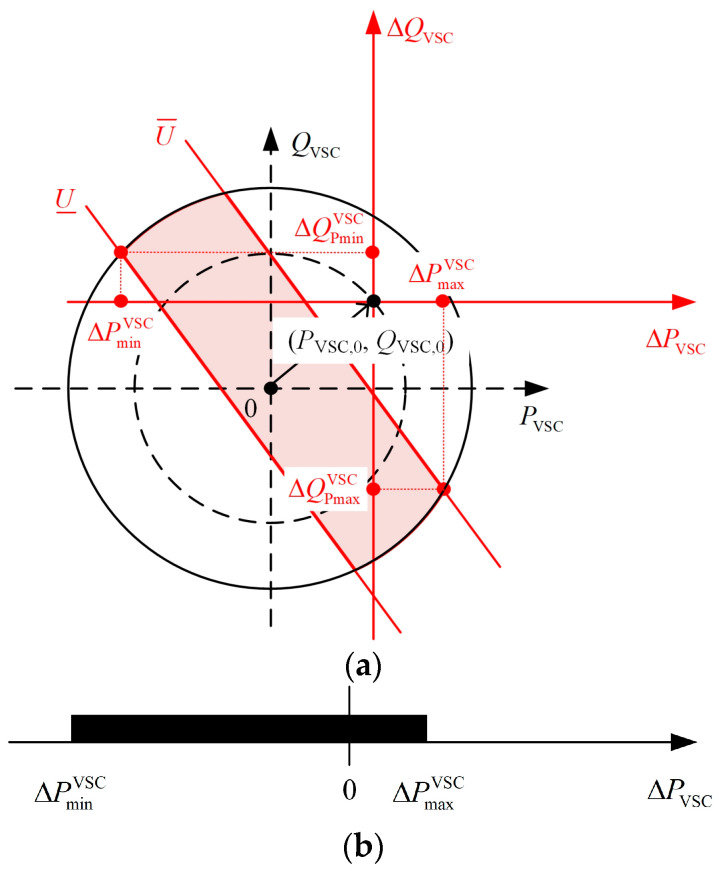
(**a**) Schematic diagram of the adjustable range of VSC output power; (**b**) schematic diagram of the adjustable range of VSC output active power.

The intersection of $\Omega_{P}$ described by Equations (6) and (7) represents the range of adjustable active power output values for the VSC. As shown in Figure 4a, with Δ*P*_VSC_ as the horizontal axis and Δ*Q*_VSC_ as the vertical axis, the region described by Equation (6) is between two parallel lines with slopes of $-S_{P}^{\mathrm{VSC}}/S_{Q}^{\mathrm{VSC}}$, and the region described by Equation (7) is a circular region with *S*_VSC_ as the radius and $\left(-P_{0},-Q_{0}\right)$ as the center. The intersection of these two regions forms the feasible range of adjustable VSC output power.

According to Figure 4a, Equations (6) and (7), the minimum and maximum value of the adjustable range of VSC output active power can be obtained, as described in Equations (8) and (9):

(8) $$\Delta P_{\min}^{\mathrm{VSC}}=\min\left\{\frac{Ak-P_{0}+C}{k^{2}+1},\frac{Ak-P_{0}+C}{k^{2}+1},\frac{Bk-P_{0}+D}{k^{2}+1},\frac{Bk-P_{0}+D}{k^{2}+1}\right\}$$

(9) $$\Delta P_{\max}^{\mathrm{VSC}}=\max\left\{\frac{Ak-P_{0}+C}{k^{2}+1},\frac{Ak-P_{0}+C}{k^{2}+1},\frac{Bk-P_{0}+D}{k^{2}+1},\frac{Bk-P_{0}+D}{k^{2}+1}\right\}$$

where $A=Q_{0}-\frac{\underline{U}-U_{0}}{S_{Q}^{\mathrm{VSC}}}$, $B=Q_{0}+\frac{\bar{U}-U_{0}}{S_{Q}^{\mathrm{VSC}}}$, $C=\sqrt{-A^{2}-2AP_{0}k-P_{0}^{2}k^{2}+S^{2}k^{2}+S^{2}}$, $D=\sqrt{-B^{2}-2BP_{0}k-P_{0}^{2}k^{2}+S^{2}k^{2}+S^{2}}$, $k=\frac{S_{P}^{\mathrm{VSC}}}{S_{Q}^{\mathrm{VSC}}}$. Moreover, as indicated by Figure 4a, the points corresponding to $\Delta P_{\min}^{\mathrm{VSC}}$ and $\Delta P_{\max}^{\mathrm{VSC}}$ are unique for each VSC. For the sake of later discussions, set $\Delta Q_{\mathrm{Pmin}}^{\mathrm{VSC}}$ and $\Delta Q_{\mathrm{Pmax}}^{\mathrm{VSC}}$, respectively, to represent the adjustments of reactive power output when the VSC output active power adjustment is $\Delta P_{\min}^{\mathrm{VSC}}$ and $\Delta P_{\max}^{\mathrm{VSC}}$.

**Figure 5:**
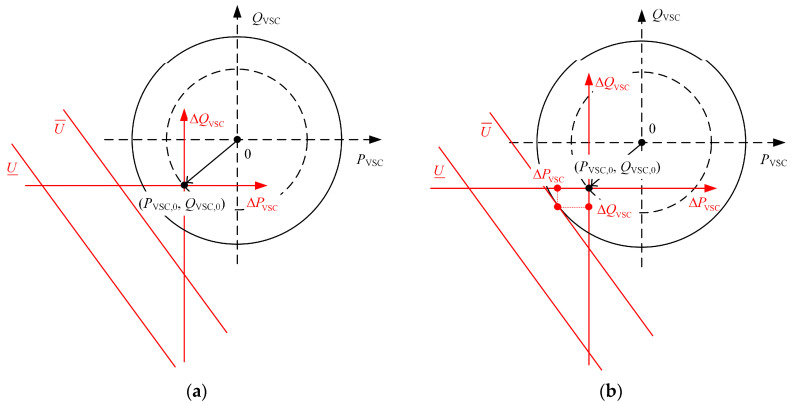
(**a**) Voltage exceeding upper limit and VSC capacity is insufficient to regulate; (**b**) VSC capacity is sufficient to regulate terminal node voltage after curtailing PV power.

Figure 4 shows that the VSC capacity is sufficient to maintain the terminal node voltage within the permissible range. However, in certain extreme situations, such as when the PV output power in a DSA is excessively high, it results in a significant voltage violation at the terminal node, as illustrated in Figure 5a. Due to the capacity limitations of the VSC, solely adjusting the power injected by the VSC to the terminal node is no longer effective in bringing the voltage back to within the allowable range.

In this extreme scenario depicted in Figure 5a, even if the VSC operates at its maximum capacity, it cannot bring the voltage back to within the allowable range. To prevent voltage violations in this case, it is necessary to actively curtail a portion of the PV output power. To minimize curtailment, the two parallel lines representing the upper and lower voltage limits should be shifted to the position shown in Figure 5b after curtailment. At this point, the circle representing the VSC capacity is just tangent to the line representing the upper voltage limit. This means that using the entire capacity of the VSC for compensating the terminal node voltage will precisely control the voltage so as not to exceed the upper limit. The following derivation outlines the minimum curtailment in this extreme situation.

In Figure 5b, the VSC operating point $\left(\Delta P_{\mathrm{VSC}},\Delta Q_{\mathrm{VSC}}\right)$ where the line representing the upper voltage limit is tangent to the circle representing the VSC capacity satisfies Equation (10):

(10) $$\Delta Q_{\mathrm{VSC}}=\frac{S_{Q}^{\mathrm{VSC}}}{S_{P}^{\mathrm{VSC}}}\cdot \Delta P_{\mathrm{VSC}}$$

After adjusting the VSC output power to make it operate at the tangent point, the terminal node voltage $\tilde{U}$ is given by Equation (11):

(11) $$\tilde{U}=U_{0}+\frac{{\left(S_{P}^{\mathrm{VSC}}\right)}^{2}+{\left(S_{Q}^{\mathrm{VSC}}\right)}^{2}}{S_{P}^{\mathrm{VSC}}}\Delta P_{\mathrm{VSC}}$$

It needs to curtail PV power to compensate for the voltage deviation between the voltage upper limit $\bar{U}$ and the voltage value $\tilde{U}$. The minimum curtailing portion of PV power *P*_cur_ can be derived as shown in Equation (12):

(12) $$P_{\mathrm{cur}}=\frac{\bar{U}-\tilde{U}}{S_{P}^{\mathrm{PV}}}=\frac{\bar{U}-U_{0}}{S_{P}^{\mathrm{PV}}}-\frac{{\left(S_{P}^{\mathrm{VSC}}\right)}^{2}+{\left(S_{Q}^{\mathrm{VSC}}\right)}^{2}}{S_{P}^{\mathrm{VSC}}\cdot S_{P}^{\mathrm{PV}}}\cdot \Delta P_{\mathrm{VSC}}$$

where $S_{P}^{\mathrm{PV}}$ represents the sensitivity coefficient between the change in injected active power of the nodes connected to PV and the change in terminal node voltage.

The adjustment of active power output corresponding to the tangent point in Figure 5b can be represented by Equation (13):

(13) $$\Delta P_{\mathrm{VSC}}=\frac{\left(\bar{U}-U_{0}\right)\cdot S_{P}^{\mathrm{VSC}}}{{\left(S_{P}^{\mathrm{VSC}}\right)}^{2}+{\left(S_{Q}^{\mathrm{VSC}}\right)}^{2}}$$

For uniform representation, the minimum values $\Delta P_{\min}^{\mathrm{VSC}}$ and maximum values $\Delta P_{\max}^{\mathrm{VSC}}$ of the adjustable active power output range of VSC for such extreme scenarios as shown in Figure 5 are defined as in Equation (14):

(14) $$\Delta P_{\min}^{\mathrm{VSC}}=\Delta P_{\max}^{\mathrm{VSC}}=\frac{\left(\bar{U}-U_{0}\right)\cdot S_{P}^{\mathrm{VSC}}}{{\left(S_{P}^{\mathrm{VSC}}\right)}^{2}+{\left(S_{Q}^{\mathrm{VSC}}\right)}^{2}}$$

For the extreme scenario where the voltage at the terminal nodes of the DSA exceeds the lower limit due to excessive load, and due to the limited capacity of the VSC, it is not possible to rely solely on the VSC to adjust the terminal voltage back to the allowed range. This scenario is similar to the voltage exceeding the upper limit due to excessive output power from the PV. Therefore, the analysis of this extreme scenario is not further elaborated.

### 3.2. The Proposed Strategy for SOP

The adjustable range of VSC output power $\Omega_{P}$ in each DSA has been derived above. This section focuses on discussing how to specify the adjustment of the output power for each VSC to achieve the goals of regulating terminal node voltages and balancing load rates among interconnected DSAs. In addition, due to the different $\Omega_{P}$ in different operating states, it may not be possible to meet simultaneously the objectives of regulating terminal node voltages and achieving load rate balancing in certain scenarios. Therefore, set the priority order for the control goals as follows: No terminal voltage violations > minimize curtailed PV power > load rate balancing among DSAs.

Since SOP itself does not generate or store active power, the adjustment amount of active power output from each VSC needs to not only fall within the given range $\Omega_{P}$ but also satisfy the energy conservation constraint, as shown in Equation (15) [26]:

(15) $$\sum_{n=1}^{N}\Delta P_{\mathrm{VSC},n}=0$$

where N represents the number of interconnected DSAs, and the subscript *n* denotes the DSA *n*, etc.

Define the range of the sum of the $\Omega_{P}$ for each DSA, as $\Omega_{P}^{\mathrm{sum}}$, as shown in Equation (16).

(16) $$\Omega_{P}^{\mathrm{sum}}=\left\{\left.\sum_{n=1}^{N}\Delta P_{\mathrm{VSC},n}\right|\sum_{n=1}^{N}\Delta P_{\min,n}^{\mathrm{VSC}}\leq \sum_{n=1}^{N}\Delta P_{\mathrm{VSC},n}\leq \sum_{n=1}^{N}\Delta P_{\max,n}^{\mathrm{VSC}}\right\}$$

For energy conservation, when $\Omega_{P}^{\mathrm{sum}}$ does not include 0, it indicates that adjusting VSC output power alone cannot satisfy the voltage constraints at the terminals of each DSA. In this case, measures such as curtailing PV power or shedding load need to be considered to change $\Omega_{P}^{\mathrm{sum}}$ to include 0. When $\Omega_{P}^{\mathrm{sum}}$ includes 0, it indicates that, within the allowable range of VSC capacity, adjusting the VSC output power can ensure that the voltages at the end of all DSAs do not exceed the limits. Further consideration can be given to achieving the control objective of load rate balance.

The proposed strategy involves real-time monitoring of the DSA operating state, computing $\Omega_{P}^{\mathrm{sum}}$, and then categorizing different operating states into four scenarios. For each scenario, a corresponding VSC output power adjustment scheme is designed to meet the control objectives.

**Figure 6:**
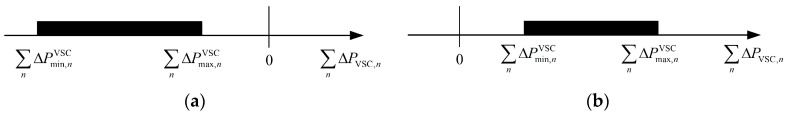
Schematic diagram of Scenario 1 corresponding to $\Omega_{P}^{\mathrm{sum}}$: (**a**) $\sum_{n=1}^{N}\Delta P_{\max,n}^{\mathrm{VSC}}<0$; (**b**) $\sum_{n=1}^{N}\Delta P_{\min,n}^{\mathrm{VSC}}>0$.

(1) Scenario 1: $\Omega_{P}^{\mathrm{sum}}$ does not include 0, i.e., $\sum_{n=1}^{N}\Delta P_{\max,n}^{\mathrm{VSC}}<0$ or $\sum_{n=1}^{N}\Delta P_{\min,n}^{\mathrm{VSC}}>0$, as shown in Figure 6.

Scenario 1, as shown in Figure 6a, indicates that under the constraint of VSC capacity, in order to prevent the voltage at the end of each DSA from exceeding the limit, the sum of active power that needs to be transferred from some DSAs is more than the sum of active power that the remaining DSAs can consume. This situation means that the SOP cannot ensure that the voltage at the end of all DSAs does not exceed the limit by transferring active power between DSAs and provide reactive power compensation. In Figure 6b, under the constraints of VSC capacity and voltage limits, the sum of active power that some DSAs need to inject is more than the sum of active power that the remaining DSAs can provide.

The two extreme cases depicted in Scenario 1 illustrate that, even if the entire capacity of VSC is used to regulate the voltage, it is still impossible to control it within the permissible range. As shown in Figure 4, curtailing PV power or shedding load changes the relative position of the two parallel lines representing the voltage limits and the VSC capacity circle. This change can alter the values of $\Omega_{P}$ for each DSA, thereby modifying $\Omega_{P}^{\mathrm{sum}}$ to include 0. Therefore, for the two extreme cases shown in Scenario 1, it is necessary to curtail PV power, shed load, and cooperate with the SOP to regulate the terminal voltage. The amounts of minimum PV output power curtailment, minimum load shedding, and VSC output power adjustment can be determined by optimization model 1, as shown in Equation (17):

(17) $$\begin{array}{l} \left(\mathrm{Model}\;1\right)\underset{\left\{\Delta P_{\mathrm{VSC},n},\Delta Q_{\mathrm{VSC},n},P_{\mathrm{cur},i},P_{\mathrm{shed},j}\right\}}{\min}\sum_{n=1}^{N}\left(\sum_{i\in \Phi_{\mathrm{PV},n}}P_{\mathrm{cur},i}+M\sum_{j\in \Phi_{\mathrm{load},n}}P_{\mathrm{shed},j}\right)\\ s.t.\;{\left(P_{0,n}+\Delta P_{\mathrm{VSC},n}\right)}^{2}+{\left(Q_{0,n}+\Delta Q_{\mathrm{VSC},n}\right)}^{2}\leq {\left(S_{\mathrm{VSC},n}\right)}^{2}\\ \underline{U}\leq U_{0,n}+\Delta P_{\mathrm{VSC},n}\cdot S_{P}^{\mathrm{VSC}}+\Delta Q_{\mathrm{VSC},n}\cdot S_{Q}^{\mathrm{VSC}}-\sum_{i\in \Phi_{\mathrm{PV},n}}P_{\mathrm{cur},i}\cdot S_{P,i}+\sum_{j\in \Phi_{\mathrm{load},n}}P_{\mathrm{shed},j}\cdot S_{P,j}\leq \bar{U}\\ \sum_{n}\Delta P_{\mathrm{VSC},n}=0\\ 0\leq P_{\mathrm{cur},i}\leq P_{\mathrm{PV},i}\\ 0\leq P_{\mathrm{shed},j}\leq P_{\mathrm{load},j} \end{array}$$

where $\Phi_{\mathrm{PV},n}$ and $\Phi_{\mathrm{load},n}$ represent the set of nodes connected to PVs and load in the DSA *n*, respectively. $P_{\mathrm{cur},i}$ and $P_{\mathrm{shed},j}$ represent the amount of PV output power curtailment at node *I* and the load shedding at node *j*, respectively. $S_{P,i}$ and $S_{P,j}$, respectively, represent the sensitivity coefficients between the adjustment of injected active power and terminal voltage at nodes *I* and *j*. $P_{\mathrm{PV},i}$ and $P_{\mathrm{load},j}$, respectively, represent the PV output power at node *I* and the load at node *j*. M is a sufficiently large number used as a coefficient for shedding load to achieve the goal of minimizing load shedding as much as possible.

By solving optimization model 1 the minimum shedding of load can be obtained while minimizing the curtailing of PV as much as possible, along with the active and reactive power adjustment values for VSC outputs in all DSAs. The VSC output power reference values for Scenario 1 are given by Equations (18) and (19):

(18) $$P_{\mathrm{VSC},n}^{\mathrm{ref},\;I}=P_{\mathrm{VSC},0,n}+\Delta P_{\mathrm{VSC},n}$$

(19) $$Q_{\mathrm{VSC},n}^{\mathrm{ref},\;I}=Q_{\mathrm{VSC},0,n}+\Delta Q_{\mathrm{VSC},n}$$

where $P_{\mathrm{VSC},n}^{\mathrm{ref},\;I}$ and $Q_{\mathrm{VSC},n}^{\mathrm{ref},\;I}$ respectively represent the reference values for active and reactive power outputs of VSC in the DSA *n* in Scenario 1.

In addition, since the reference values for VSC output power obtained from optimization model 1 have already reached the upper limit of VSC capacity, this precisely ensures that all region voltages are within limits. That is, within the capacity range of VSCs in each DSA, their operating points are unique. Thus, for Scenario 1, this extreme case does not allow for further pursuit of the goal of balancing the load rate among DSAs while keeping the voltages within limits; or it requires additional curtailing PV power or shedding load, which is evidently not rational. Therefore, Scenario 1 only considers finding the operating state with the minimum curtailing PV power or shedding the load that ensures all voltages at the end of the DSAs remain within limits.

**Figure 7:**
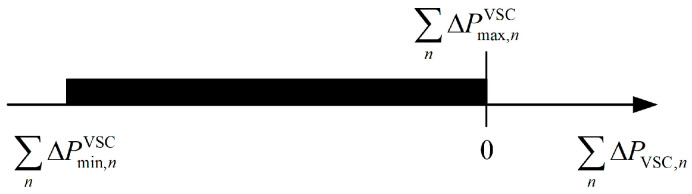
Schematic diagram of Scenario 2 corresponding to $\Omega_{P}^{\mathrm{sum}}$.

(2) Scenario 2: The upper limit of $\Omega_{P}^{\mathrm{sum}}$ is set to 0, i.e., $\sum_{n=1}^{N}\Delta P_{\max,n}^{\mathrm{VSC}}=0$, as shown in Figure 7.

Scenario 2, as shown in Figure 7, under the constraints of VSC capacity and the upper and lower limits of the voltage at the end of each DSA, depicts the minimum amount of the sum of active power that some DSAs need to transfer to the others, which is equal to the maximum amount of active power that the remaining DSAs can consume. Moreover, in this scenario, the VSCs must adjust their output active power according to their maximum value of $\Omega_{P}$. Because only this adjustment can satisfy the energy conservation constraint, this means the operating point of the VSC is unique in this scenario. The reference values for the active and reactive power output of the VSC in the DSA *n* in Scenario 2 are $P_{\mathrm{VSC},n}^{\mathrm{ref},\;\mathrm{II}}$ and $Q_{\mathrm{VSC},n}^{\mathrm{ref},\;\mathrm{II}}$, as shown in Equations (20) and (21):

(20) $$P_{\mathrm{VSC},n}^{\mathrm{ref},\;\mathrm{II}}=P_{\mathrm{VSC},0,n}+\Delta P_{\max,n}^{\mathrm{VSC}}$$

(21) $$Q_{\mathrm{VSC},n}^{\mathrm{ref},\;\mathrm{II}}=Q_{\mathrm{VSC},0,n}+\Delta Q_{P\max,n}^{\mathrm{VSC}}$$

**Figure 8:**
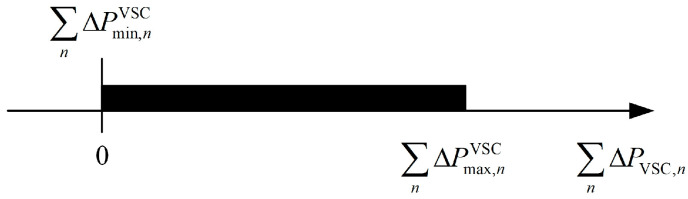
Schematic diagram of Scenario 3 corresponding to $\Omega_{P}^{\mathrm{sum}}$.

(3) Scenario 3: The lower limit of $\Omega_{P}^{\mathrm{sum}}$ is equal to 0, i.e., $\sum_{n=1}^{N}\Delta P_{\min,n}^{\mathrm{VSC}}=0$, as shown in Figure 8.

Similar to Scenario 2, Scenario 3 as shown in Figure 8, under the constraints of VSC capacity and the upper and lower limits of the voltage at the end of each DSA, depicts the maximum amount of the sum of active power that some DSAs need to transfer to the others, which is equal to the minimum amount of active power that the remaining DSAs can consume. In this case, each VSC adjusts the injected active power according to their minimum value of $\Omega_{P}$, and sets the injected reactive power based on the corresponding adjustment value. The reference values for the active and reactive power output of the VSC in the DSA *n* in Scenario 3 are $P_{\mathrm{VSC},n}^{\mathrm{ref},\;\mathrm{III}}$ and $Q_{\mathrm{VSC},n}^{\mathrm{ref},\;\mathrm{III}}$, as shown in Equations (22) and (23):

(22) $$P_{\mathrm{VSC},n}^{\mathrm{ref},\;\mathrm{III}}=P_{\mathrm{VSC},0,n}+\Delta P_{\min,n}^{\mathrm{VSC}}$$

(23) $$Q_{\mathrm{VSC},n}^{\mathrm{ref},\;\mathrm{III}}=Q_{\mathrm{VSC},0,n}+\Delta Q_{P\min,n}^{\mathrm{VSC}}$$

**Figure 9:**
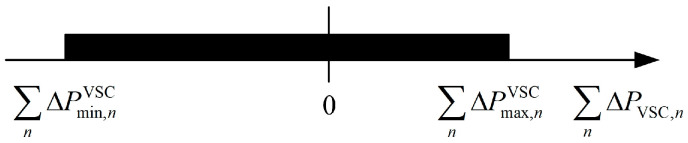
Schematic diagram of Scenario 4 corresponding to $\Omega_{P}^{\mathrm{sum}}$.

(4) Scenario 4: $\Omega_{P}^{\mathrm{sum}}$ includes 0, i.e., $\sum_{n=1}^{N}\Delta P_{\min,n}^{\mathrm{VSC}}<0<\sum_{n=1}^{N}\Delta P_{\max,n}^{\mathrm{VSC}}$, as shown in Figure 9.

As shown in Figure 9, Scenario 4 indicates that the interconnected DSAs can not only achieve the prevention of voltage violations at the end of each DSA by mutually transferring active power, but the amount of transferred active power can also have multiple feasible values. This implies that, on the basis of maintaining voltage within the allowed limits, an optimal power transfer scheme can be selected, balancing the load rates among DSAs.

The adjustment values of VSC output power for each DSA in Scenario 4 are obtained from the optimization model 2 as shown in Equation (24):

(24) $$\begin{array}{l} \left(\mathrm{Model}\;2\right)\underset{\left\{\Delta P_{\mathrm{VSC},n},\Delta Q_{\mathrm{VSC},n}\right\}}{\min}\sum_{n=1}^{N}\Delta \Phi_{n}\\ s.t.\;\Delta \Phi_{n}=\left|\Phi_{n}-\bar{\Phi }\right|\\ \Phi_{n}=\frac{P_{n}^{\mathrm{trans}}}{S_{n}^{\mathrm{trans}}}\\ \bar{\Phi }=\frac{1}{N}\sum_{n=1}^{N}\frac{P_{n}^{\mathrm{trans}}}{S_{n}^{\mathrm{trans}}}\\ {\left(P_{0,n}+\Delta P_{\mathrm{VSC},n}\right)}^{2}+{\left(Q_{0,n}+\Delta Q_{\mathrm{VSC},n}\right)}^{2}\leq {\left(S_{\mathrm{VSC},n}\right)}^{2}\\ \underline{U}\leq U_{n,0}+\Delta P_{\mathrm{VSC},n}\cdot S_{P}^{\mathrm{VSC}}+\Delta Q_{\mathrm{VSC},n}\cdot S_{Q}^{\mathrm{VSC}}\leq \bar{U}\\ \Delta P_{\min,n}^{\mathrm{VSC}}\leq \Delta P_{\mathrm{VSC},n}\leq \Delta P_{\max,n}^{\mathrm{VSC}}\\ \sum_{n}\Delta P_{\mathrm{VSC},n}=0\\ P_{n}^{\mathrm{trans}}=P_{0,n}^{\mathrm{trans}}-\Delta P_{\mathrm{VSC},n} \end{array}$$

where $S_{n}^{\mathrm{trans}}$, $P_{0,n}^{\mathrm{trans}}$, and $P_{n}^{\mathrm{trans}}$ represent the transformer capacity, the active power flow before and after adjusting the VSC output power through the transformer in the DSA *n*, respectively. $\bar{\Phi }$, $\Phi_{n}$, and $\Delta \Phi_{n}$ represent the average load rate after adjusting VSC output power, the load rate of the DSA *n*, and its load rate imbalance degree.

The reference values for the active and reactive power output of the VSC in the DSA *n* in Scenario 4 are $P_{\mathrm{VSC},n}^{\mathrm{ref},\;\mathrm{IV}}$ and $Q_{\mathrm{VSC},n}^{\mathrm{ref},\;\mathrm{IV}}$, as shown in Equations (25) and (26):

(25) $$P_{\mathrm{VSC},n}^{\mathrm{ref},\;\mathrm{IV}}=P_{\mathrm{VSC},0,n}+\Delta P_{\mathrm{VSC},n}$$

(26) $$Q_{\mathrm{VSC},n}^{\mathrm{ref},\;\mathrm{IV}}=Q_{\mathrm{VSC},0,n}+\Delta Q_{\mathrm{VSC},n}$$

**Figure 10:**
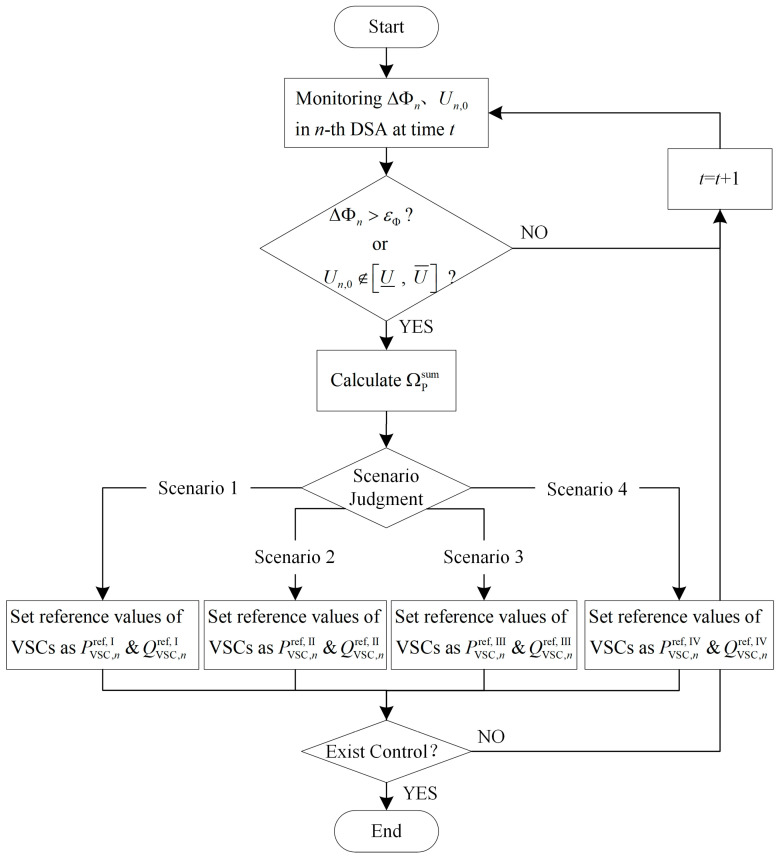
Process flowchart of proposed control strategy.

The complete process of the proposed strategy is illustrated in Figure 10. At time *t*, it begins with monitoring the load rate imbalance degree $\Delta \Phi_{n}$ and voltage values at the end of feeders $U_{n,0}$ in each DSA. Next, it checks whether the sum of load rate imbalance degree in all DSAs exceeds the set range $\varepsilon_{\Phi }$ or if any DSA experiences a voltage violation. If no exceedance is detected, the output power of the VSCs will not be adjusted. However, in case of exceedance, it calculates $\sum_{n=1}^{N}\Delta P_{\min,n}^{\mathrm{VSC}}$ and $\sum_{n=1}^{N}\Delta P_{\max,n}^{\mathrm{VSC}}$, and then determines the scenario. Following the instructions provided earlier for different scenarios, it adjusts the VSC output power, curtailing PV power or shedding load accordingly.

## 4. Case Study

### 4.1. Settings of Cases

**Figure 11:**
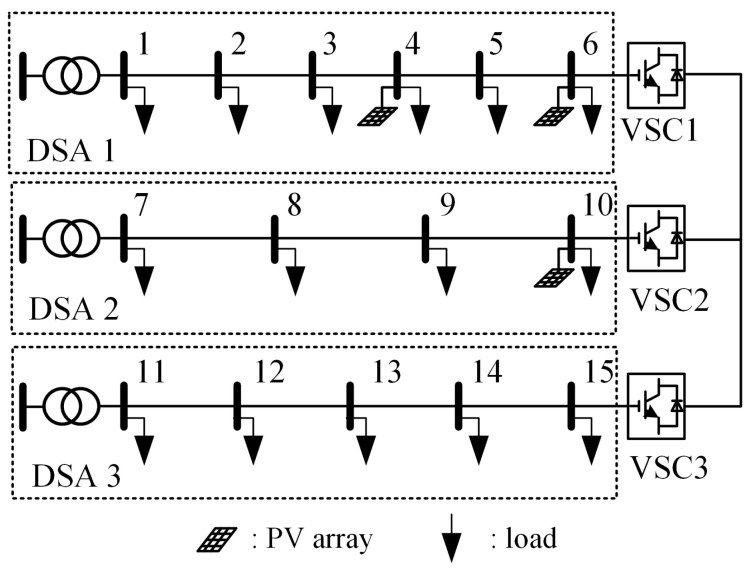
Topology of the interconnected three-DSAs model in case study.

To validate the effectiveness of the proposed strategy, a flexible interconnected three-DSAs model was established in MATLAB/Simulink, as shown in Figure 11. The rated voltage on the AC side *U*_N_ is 380 V, with voltage limits set at 1.07 *U*_N_ and 0.93 *U*_N_. The transformers in the three DSAs have rated capacities of 150 kVA, 200 kVA, and 100 kVA, respectively. The capacities of the three VSCs are all set to 75 kVA.

**Figure 12:**
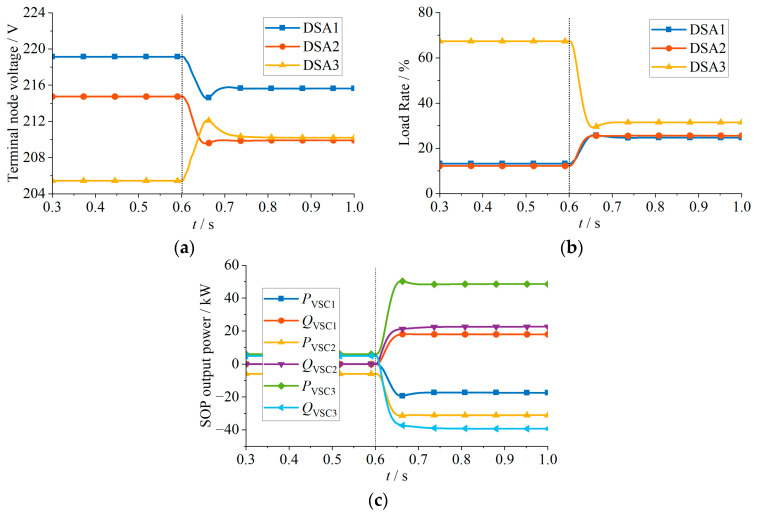
Simulation results of Case 1: (**a**) Terminal node voltage; (**b**) load rate; (**c**) SOP output power.

**Figure 13:**
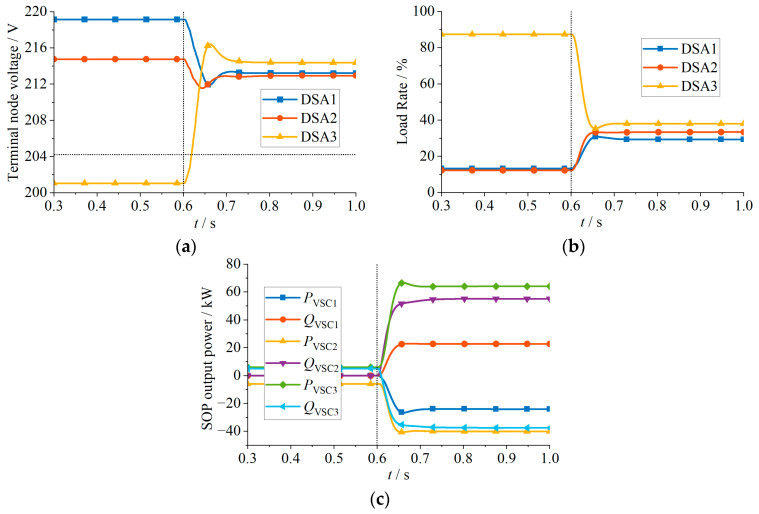
Simulation results of Case 2: (**a**) Terminal node voltage; (**b**) load rate; (**c**) SOP output power.

**Figure 14:**
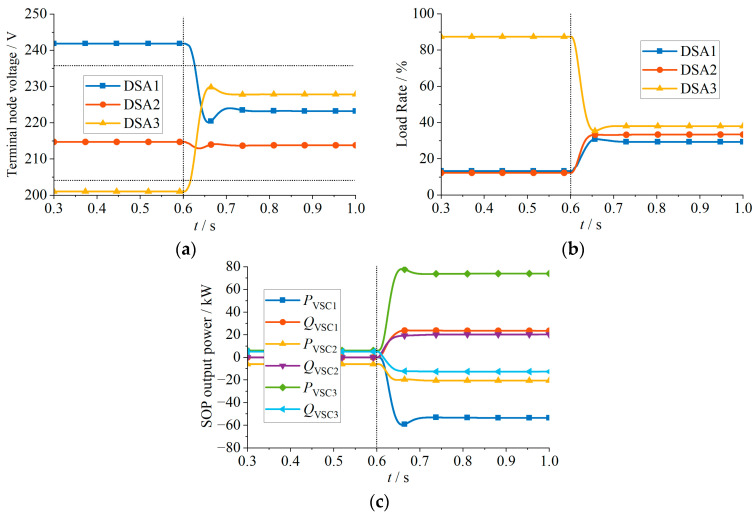
Simulation results of Case 3: (**a**) Terminal node voltage; (**b**) load rate; (**c**) SOP output power.

**Figure 15:**
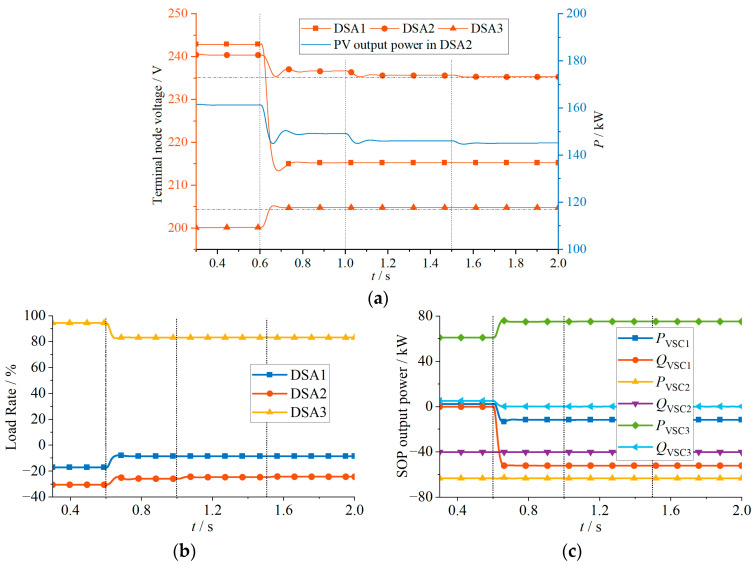
Simulation results of Case 4: (**a**) Terminal node voltage and the output power of PV in DSA2; (**b**) load rate; (**c**) SOP output power.

The following four simulation cases are designed, and in each case, the proposed strategy was activated at 0.6 s. The simulation results are illustrated in Figure 12, Figure 13, Figure 14 and Figure 15.

Case 1: Terminal node voltages of the three DSAs remain within limits, but there is a significant difference in load rates.

Case 2: Terminal node voltage exceeds limits in a single DSA, and the VSC capacity is sufficient to regulate.

Case 3: Terminal node voltages exceed limits in two DSAs, and the VSC capacity is sufficient to regulate.

Case 4: Terminal node voltages exceed limits in all three DSAs, but the VSC capacity is insufficient to regulate.

### 4.2. Results and Discussion

The simulation results for Case 1 are shown in Figure 12 and Table 1. At the beginning, the load rates among the three DSAs are severely imbalanced. At 0.6 s, the proposed strategy is activated to adjust the VSC output power. Specifically, the strategy controls DSA 1 and 2, with lighter loads, to transfer 17.35 kW and 25.14 kW of active power to DSA 3, achieving balanced load rates of around 25% for all three DSAs. As illustrated in Figure 12c, the VSCs in DSA 1 and 2 increase the injected reactive power, ensuring that terminal node voltages remain within the permissible range. As shown in Figure 12b, the slightly higher load rate of DSA 3 compared to DSA 1 and DSA 2 after adjusting the VSC output power is because VSC 3 increases the active power injection while, at the same time, to prevent the end-node voltage from exceeding the upper limit, it increases the consumption of inductive reactive power. This results in increased network losses in the feeder, leading to a slightly higher load rate in DSA 3. However, it is not necessary for the load rates of the DSAs to be exactly equal; it is sufficient to avoid severely imbalanced scenarios.

The simulation results for Case 2 are shown in Figure 13 and Table 2. At the beginning, the terminal node voltage of DSA 3 violates the lower limit, reaching 201.03 V. At 0.6 s, the proposed strategy is activated, as shown in Figure 13c. DSA 1 and 2 transfer 24.04 kW and 34.05 kW of active power to DSA 3, raising the terminal voltage to 214.35 V, within the permissible range. Moreover, since the VSC capacity is sufficient, Case 2 falls into Scenario 4 described earlier, where adjusting the VSC output power ensures that all terminal node voltages meet the constraints. Furthermore, it can further reduce the load rate deviation among DSAs, as illustrated in Figure 13b, where the load rates of the three DSAs become approximately equal after the adjustment.

The simulation results for Case 3 are shown in Figure 14 and Table 3. At the beginning, the terminal voltages of DSA 1 and DSA 3 violate the upper and lower voltage limits, reaching 241.85 V and 201.03 V. At 0.6 s, the proposed strategy is activated, as shown in Figure 14c, and DSA 1 and 2 transfer 57.34 kW and 11.35 kW of active power to DSA 3. As illustrated in Figure 14a,b, after adjusting the VSC output power, the terminal node voltages in DSA 1 and 3 return to within the upper and lower limits. Additionally, the load rates among the three DSAs become approximately balanced. The PV output power in DSA 1, which is originally unable to be locally consumed, is transferred to DSA 3 for joint consumption. It is effective in improving the PV consumption rate.

Case 4 is an extreme case, and its simulation results are shown in Figure 15 and Table 4. DSA 1 and 3 have the same initial conditions as in Case 3. In DSA 2, due to excessive PV output power, the terminal node voltage violates the upper limit severely, reaching 242.87 V. As shown in Figure 15c, before adjusting the output power of VSC 2, the VSC was already in a full-capacity operation state and could not further reduce the terminal node voltage by changing the output power. So, DSA 2 can only reduce the terminal voltage by curtailing the PV output power. At 0.6 s, the proposed strategy is activated. DSA 1 transfers some of the unconsumed PV output power to DSA 3 for joint consumption, and the reactive power compensation of the VSCs is adjusted to ensure that the terminal node voltages of DSAs 1 and DSA 3 return to within the upper and lower limits. DSA 2, due to insufficient VSC capacity to adjust the terminal voltage, reduces the voltage by curtailing the PV output power. As shown in Figure 15a, at 0.6 s, the curtailing PV output power calculated based on the proposed strategy cannot directly bring the terminal voltage of DSA 2 back within the upper and lower limits, and there is a slight deviation from the upper limit. This is because the voltage–reactive power sensitivity coefficient mentioned before, derived based on some approximate assumptions, is an approximate value. In the extreme case with large voltage deviations, the power adjustment calculated based on the approximate sensitivity coefficient cannot completely achieve the expected voltage adjustment, resulting in little deviation. In this case, the proposed strategy can be repeated in the next control period to correct this deviation, ultimately satisfying the voltage constraint. In Case 4, the strategy is executed again at 1.0 s and 1.5 s, as shown in Figure 15a. After two adjustments to the PV output power at 1.0 s and 1.5 s, the terminal node voltage of DSA 2 also returns to within the upper and lower limits.

## 5. Conclusions

This paper proposes a novel control strategy for SOP to address the issues of the voltage violations at the end of feeders and the unbalanced load rates among DSAs. This strategy takes into account the current situation where sensor devices are not sufficiently equipped in low-voltage DSAs. It achieves control objectives based solely on local information from the SOP and the information regarding the transformer load rates, thereby avoiding the need to establish a global sensor and communication network. Simulation results demonstrate that the strategy effectively addresses voltage violations and unbalanced load rates caused by the inability to locally consume PV output power or handle heavy loads in DSAs. It could play a significant role in improving RES penetration and the operational economy of DSAs. What needs attention is when the proposed strategy applies to real-time scenarios as it has certain limitation. In the extreme case with large voltage deviations, since the voltage–active power sensitivity coefficient used in this strategy is an approximate value, it may be difficult to achieve completely the expected voltage adjustment in one cycle. It needs more control cycles to correct this deviation for this strategy. Future research will explore how to expand this control strategy to make it applicable to hybrid AC/DC distribution networks.

## Figures and Tables

**Table 1:** Simulation results of Case 1.

DSAs	Terminal Node Voltage (V)		Load Rate (%)		SOP Output Active Power (kW)	
	Before	After	Before	After	Before	After
DSA 1	219.14	215.63	13.27	24.73	−0.058	−17.407
DSA 2	214.74	209.91	12.27	25.58	−6.000	−31.145
DSA 3	205.45	210.21	67.34	31.46	6.001	48.494

**Table 2:** Simulation results of Case 2.

DSAs	Terminal Node Voltage (V)		Load Rate (%)		SOP Output Active Power (kW)	
	Before	After	Before	After	Before	After
DSA 1	219.14	213.21	13.27	29.38	−0.058	−24.093
DSA 2	214.74	212.92	12.27	33.37	−6.000	−40.047
DSA 3	201.04	214.35	87.38	38.00	6.001	64.081

**Table 3:** Simulation results of Case 3.

DSAs	Terminal Node Voltage (V)		Load Rate (%)		SOP Output Active Power (kW)	
	Before	After	Before	After	Before	After
DSA 1	241.84	223.18	−16.11	12.64	−0.062	−53.464
DSA 2	214.74	213.80	12.27	20.06	−6.000	−20.468
DSA 3	201.04	227.84	87.38	29.66	6.000	73.868

**Table 4:** Simulation results of Case 4.

	DSAs	Before Adjustment	First Adjustment (0.6 s)	Second Adjustment (1.0 s)	Third Adjustment (1.5 s)
Terminal node voltage (V)	DSA 1	242.87	215.20	215.19	215.19
	DSA 2	240.42	236.56	235.59	235.26
	DSA 3	200.11	204.76	204.76	204.76
Load Rate (%)	DSA 1	−17.15	−8.53	−8.52	−8.52
	DSA 2	−30.52	25.93	−24.78	−24.38
	DSA 3	94.48	83.20	83.20	83.20
SOP output active power (kW)	DSA 1	2.271	−11.721	−11.724	−11.723
	DSA 2	−63.338	−63.339	−63.338	−63.337
	DSA 3	61.000	74.999	75.000	75.000

## Data Availability

Data are contained within the article.

## References

[1] Wang Y., Ai X., Tan Z., Yan L., Liu S. (2015). Interactive dispatch modes and bidding strategy of multiple virtual power plants based on demand response and game theory. IEEE Trans. Smart Grid.

[2] Jianqiao Z., Jianwen Z., Dongmin X., Aozhe Z., Weipeng Z., Xu C. (2023). Terminal voltage overlimit regulation of low-voltage distribution network based on coordinated active and reactive power control of soft open point. Autom. Electr. Power Syst..

[3] Yichao D., Shouxiang W., Bingke Y. (2019). Review on evaluation methods and improvement techniques of dg hosting capacity in distribution network. Power Syst. Technol..

[4] Olivier F., Aristidou P., Ernst D., Van Cutsem T. (2016). Active management of low-voltage networks for mitigating overvoltages due to photovoltaic units. IEEE Tran. Smart Grid.

[5] Yu Y., Fushuan W., Xinglong Z., Licheng W., Cong W., Yichun G. (2023). Research review of voltage cooperative control in distribution system with high photovoltaic penetration. Electr. Power Autom. Equip..

[6] Cheng Q., Xincong L., Minhao X., Jianwen Z., Jianqiao Z., Gang S. (2022). End-line voltage control of flexible interconnected distribution grid based on Back-to-Back voltage source converter. Renew. Energy Resour..

[7] Wenling L., Zhipeng L., Haitao L. (2023). An overview of morphological development and operation control technology of power electronics dominated distribution area. Proc. CSEE.

[8] Haitao L., Xinzhou D., Xiong X., Yini X. (2023). Energy interconnection and energy microcirculation system of terminal power grid based on low-voltage flexible DC. Autom. Electr. Power Syst..

[9] Cao W., Wu J., Jenkins N., Wang C., Green T. (2016). Benefits analysis of soft open points for electrical distribution network operation. Appl. Energy.

[10] Jafari M.R., Parniani M., Ravanji M.H. (2022). Decentralized control of OLTC and PV inverters for voltage regulation in radial distribution networks with high PV penetration. IEEE Trans. Power Deliv..

[11] Zhang Z.J., Zhang Y.D., Yue D., Dou C.X., Ding L., Tan D.Y. (2022). Voltage regulation with high penetration of low-carbon energy in distribution networks: A source-grid-load-collaboration-based perspective. IEEE Trans. Industr. Inform..

[12] Krein P.T., Galtieri J.A. (2021). Active management of photovoltaic system variability with power electronics. IEEE Trans. Emerg. Sel. Top. Power Electron..

[13] Cao W., Wu J., Jenkins N., Wang C., Green T. (2016). Operating principle of soft open points for electrical distribution network operation. Appl. Energy.

[14] Li P., Ji H., Wang C., Zhao J., Song G., Ding F., Wu J. (2017). Coordinated control method of voltage and reactive power for active distribution networks based on soft open point. IEEE Trans. Sustain. Energy.

[15] Li P., Ji H., Song G., Yao M., Wang C., Wu J. (2019). A combined central and local voltage control strategy of soft open points in active distribution networks. Energy Procedia.

[16] Ji H., Yu H., Song G., Li P., Wang C., Wu J. (2019). A decentralized voltage control strategy of soft open points in active distribution networks. Energy Procedia.

[17] Li P., Ji H., Yu H., Zhao J., Wang C., Song G., Wu J. (2019). Combined decentralized and local voltage control strategy of soft open points in active distribution networks. Appl. Energy.

[18] Zhu G., Zhang Y., Ge L., Wang L. (2021). Multi-timescale voltage optimization of flexible interconnected distribution network with self-energy storage. Autom. Electr. Power Syst..

[19] Lu X.J., Zhou J., Omer S. (2023). Two-layer operation optimization of concentrated solar power with thermal energy storage system and soft open point. Int. J. Electr. Power Energy Syst..

[20] Su Y., Teh J. (2023). Two-stage optimal dispatching of AC/DC hybrid active distribution systems considering network flexibility. J. Mod. Power Syst. Clean Energy.

[21] Zheng Y., Song Y., Hill D.J. (2020). A general coordinated voltage regulation method in distribution networks with soft open points. Int. J. Electr. Power Energy Syst..

[22] Bo Z., Wei T., Yongxiang C., Yue W., Lu C. (2018). Collaborative configuration of energy storage systems and communication networks for accommodation of high-penetration residential PVs. Grid Technol..

[23] Yang Z., Yang F., Min H., Shen Y., Tang X., Hong Y., Qin L. (2023). A local control strategy for voltage fluctuation suppression in a flexible interconnected distribution station area based on soft open point. Sustainability.

[24] Cixuan C. (2016). Fundamentals of Electrical Engineering.

[25] Zheng X., Hai C. (2007). Review and applications of VSC HVDC. High Volt. Technol..

[26] Niancheng Z., Maofan Z., Xiaoxiao M., Jianquan L., Qianggang W., Yongjie L. (2023). Control and stability analysis of power transmission between microgrids based on back-to-back converter interconnection. Autom. Electr. Power Syst..

